# Relationship of choroidal thickness and axial length with posterior vitreous detachment in patients with high myopia

**DOI:** 10.1038/s41598-022-08101-7

**Published:** 2022-03-08

**Authors:** Akiko Hanyuda, Hidemasa Torii, Ken Hayashi, Atsuro Uchida, Kiwako Mori, Erisa Yotsukura, Mamoru Ogawa, Kazuno Negishi, Toshihide Kurihara, Kazuo Tsubota

**Affiliations:** 1 Department of Ophthalmology, Keio University School of Medicine, Shinjuku-ku, Tokyo Japan; 2 Laboratory of Photobiology, Keio University School of Medicine, 35 Shinanomachi, Shinjuku-ku, Tokyo 160-8582 Japan; 3 Hayashi Eye Hospital, Fukuoka, Japan; 4 Tsubota Laboratory, Inc., Shinjuku-ku, Tokyo 160-0016 Japan

**Keywords:** Diseases, Medical research, Risk factors

## Abstract

Although accumulating evidence suggests a higher prevalence of posterior vitreous detachment (PVD) in highly myopic eyes, the relation between ocular biometric features and PVD stages in such eyes remains unclear. Therefore, we enrolled 170 patients with high myopia (axial length ≥ 26.0 mm) to investigate the status of PVD regarding subfoveal choroidal thickness and axial length. Utilising swept-source optical coherence tomography, we classified the PVD status into five stages. The distribution of PVD grades increased as the choroidal thickness decreased and axial length increased (P < 0.01). On adjusting for age and sex, decreased choroidal thickness and increased axial length were associated with more advanced PVD stages: odds ratios with the highest vs. lowest groups were 0.31 (95% confidence interval [CI] 0.09–1.01; P_trend_ = 0.009) for choroidal thickness and 5.16 (95% CI 1.34–19.80; P_trend_ = 0.002) for axial length. The inverse association between choroidal thickness and PVD status seemed stronger in women than in men (P_interaction_ = 0.05). In conclusion, we firstly observed a significant trend of decreased choroidal thickness, along with increased axial length, with increased grade of PVD, particularly among women with highly myopic eyes, suggesting that advanced morphological myopic changes contribute to PVD in middle-aged adults.

## Introduction

Posterior vitreous detachment (PVD), which is defined as the separation of the vitreous cortex from the inner limiting membrane of the retina, is an age-related change in the human eyes with a prevalence exceeding 60% in those aged 70 years and older^1–3^. PVD has been associated with serious ocular complications, including retinal tears and subsequent retinal detachment^1–3^. Although the onset and progression of PVD have been largely elusive, several risk factors, including age^4^, female sex^5^, and myopia^6^, have been recognised.

The onset of vitreous liquefaction and PVD is earlier in eyes with high myopia than in those without myopia^2,5,7^. The advent of swept-source optical coherence tomography (SS-OCT), which can visualise deeper tissues in highly myopic eyes^8–10^, has spurred interest in investigating the morphological changes in the posterior vitreous and exploring the relationship between myopia and PVD. A case–control study using SS-OCT suggested a significantly earlier onset of partial and complete PVDs in patients with high myopia than in controls^11^. Further, another observational study using ultra-widefield SS-OCT found various abnormal PVDs that were specific to pathological myopia^12^. More recently, an age- and sex-matched case–control study suggested that the PVD stage was more advanced in eyes with high myopia than in those without high myopia^13^. Accordingly, high myopia characterised by increased axial length is related to the early onset of PVD; however, the relationship between other ocular features related to high myopia and PVD remains unclear.

Accumulating evidence suggests that reduced choroidal thickness in subjects with high myopia and decreased macular choroidal thickness is a significant prognostic factor for visual impairment^14,15^. Several studies have suggested that choroidal thinning occurs at an early stage of myopia progression^16–18^. Furthermore, a recent 1-year longitudinal study among school children suggested that choroidal thinning and axial elongation were independently associated with a myopic shift (i.e. at least a − 0.5 D decrease in spherical equivalent refraction [SER])^19^. These findings indicate that choroidal thickness, along with axial length, is an important biometric factor in young patients with myopia.

Accordingly, we hypothesised that middle-aged patients with more advanced myopic changes characterised by thinner choroids have higher PVD stages in high myopia. To assess this hypothesis, we precisely classified the PVD stages (e.g. paramacular, perifoveal, peripapillary, and complete)^20^ using SS-OCT and explored the association of choroidal thickness, axial length, and other ocular biometric factors with various PVD stages.

## Results

### Baseline characteristics according to partial versus complete PVD

**Figure 1:**
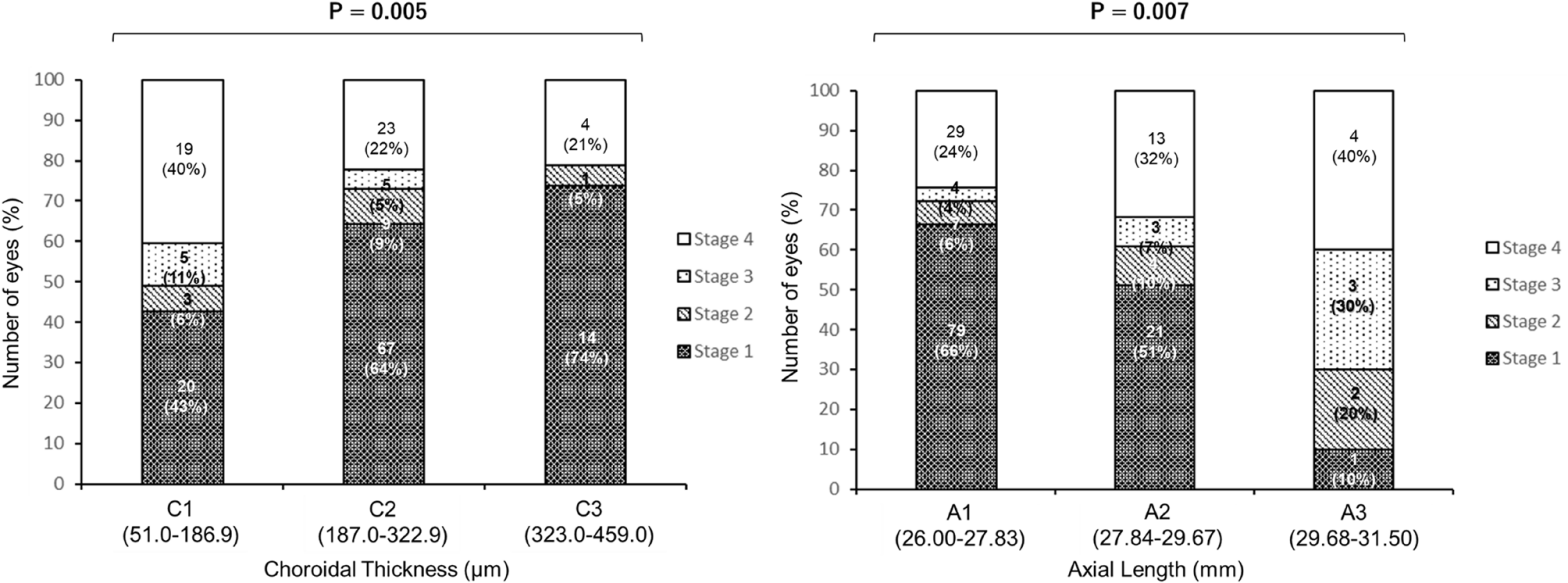
The distribution of PVD stages in relation to choroidal thickness and axial length among highly myopic eyes. The PVD stage was more progressed as decreased choroidal thickness and increased axial length.

We enrolled 170 eyes of 170 patients (46 with complete PVD and 124 with partial PVD), who were consecutive hospital visitors from 31 July 2019 to 30 September 2019 (Table 1). Among 124 patients with partial PVD, 101 (81.5%), 13 (10.5%), and 10 (8.1%) had stage 1, 2, and 3 PVDs, respectively. The mean axial length and choroidal thickness were 27.4 ± 1.1 mm and 223.2 ± 76.7 μm, respectively, in patients with partial PVD and 27.9 ± 1.3 mm and 197.7 ± 75.6 μm, respectively, in those with complete PVD. Compared to patients with partial PVD, those with complete PVD were more likely to be older, have increased axial length, worse corrected distance visual acuity (CDVA), lower SE, and decreased choroidal thickness. The proportion of sex and central retinal thickness did not differ between the partial and complete PVD groups. As shown in Fig. 1, increased axial length and decreased choroidal thickness were significantly associated with higher PVD stages (P < 0.01).

**Table 1:** Baseline demographic and clinical features in complete vs. partial PVD.

Variables	Complete PVD (n = 46)	Partial PVD (n = 124)	P value^b^
**Demographic and clinical features**^**a**^			
Mean age in years (SD)	47.1 (8.4)	36.5 (10.3)	**< 0.001**
20–29 y, n (%)	2 (4.4)	38 (30.7)	
30–39 y, n (%)	7 (15.2)	39 (31.5)	
40–49 y, n (%)	19 (41.3)	33 (26.6)	
50–59 y, n (%)	18 (39.1)	14 (11.3)	
Sex, n (%)			0.40
Male	23 (50.0)	71 (57.3)	
Spherical equivalent, diopter (SD)	− 11.4 (5.1)	− 8.68 (4.2)	**< 0.001**
Axial length, mm (SD)	27.9 (1.3)	27.4 (1.1)	**0.01**
Central retinal thickness, μm (SD)	217.4 (23.7)	217.4 (20.0)	0.99
Choroidal thickness, μm (SD)	197.7 (75.6)	223.2 (76.7)	0.05
LogMAR CDVA, (SD)	− 0.25 (0.38)	− 0.04 (0.18)	**< 0.001**
PVD stage, n (%)			
Stage 1	NA	101 (81.5)	
Stage 2	NA	13 (10.5)	
Stage 3	NA	10 (8.1)	
Stage 4	46 (100)	NA	

*CDVA* corrected distance visual acuity, *logMAR* logarithm of minimal of angle of resolution, *PVD* posterior vitreous detachment, *SD* standard deviation.

P values less than the statistically significant level (= 0.05) are marked in bold.

^a^Values are presented as means (SD) for continuous variables and percentages for categorical variables.

^b^Unpaired t-tests for continuous variables and χ^2^ tests for categorical variables were used to test for statistical significance.

### Correlations between ocular biometric factors and PVD stages

Partial correlation coefficients between PVD stages and ocular parameters in the stratum of the two age groups are summarised in Table 2. In the pooled analyses, PVD stages were positively associated with age (P < 0.001) and axial length (P < 0.001) and negatively associated with choroidal thickness (P = 0.008) and SE (P < 0.001). In the stratified analysis by age, the positive relationship of axial length and the suggestive negative relationship of choroidal thickness with PVD stages were consistently observed across the different age groups.

**Table 2:** Partial correlation analysis between PVD stages and ocular parameters in stratum of age groups.

Variable	All (n = 170)		20–< 40 years (n = 86)		40–< 60 years (n = 84)	
	r	P value	r	P value	R	P value
Age (years)	**0.43**	**< 0.001**	0.20	0.06	**0.25**	**0.02**
Sex (ref: male)	− 0.09	0.22	− 0.11	0.31	− 0.10	0.34
Axial length (mm)	**0.29**	**< 0.001**	**0.34**	**0.001**	**0.24**	**0.02**
Choroidal thickness (μm)	− **0.20**	**0.008**	− 0.20	0.06	− **0.23**	**0.04**
SE	− **0.35**	**< 0.001**	− **0.43**	**< 0.001**	− **0.32**	**0.004**

Reference, *SE* spherical equivalent.

P values less than the statistically significant level (= 0.05) are marked in bold.

### Relationship between choroidal thickness and axial length with PVD

We observed a positive relationship of axial length and an inverse relationship of choroidal thickness with the presence of complete PVD (Table 3). In the univariable analysis, the odds ratios (ORs) of complete PVD with the highest vs. lowest category were 0.39 (95% confidence interval [CI] 0.10–1.53; P_trend_ = 0.03) for choroidal thickness and 2.07 (0.55–7.85; P_trend_ = 0.01) for axial length. After adjusting for age and sex, similar associations were observed in the ORs: those with the highest vs. lowest category were 0.41 (95% CI 0.10–1.65; P_trend_ = 0.06) for choroidal thickness and 2.40 (95% CI 0.54–10.6; P_trend_ = 0.06) for axial length. This relationship was attenuated when we additionally adjusted for axial length/choroidal thickness.

**Table 3:** Univariable and multivariable-adjusted logistic regression analyses of choroidal thickness in relation to the presence of complete PVD (n = 46) (vs. partial PVD [n = 124])^‡^.

	Univariable OR (95% CI)	P value	Age-/sex-adjusted OR (95% CI)*	P value	Multivariable-adjusted OR (95% CI)^†^	P value
**Choroidal thickness**						
C1 (n = 47)	1.0 (ref)	P_trend_ = **0.03**	1.0 (ref)	P_trend_ = 0.06	1.0 (ref)	P_trend_ = 0.46
C2 (n = 104)	**0.46 (0.22–0.93)**		**0.40 (0.17–0.93)**		0.63 (0.24–1.64)	
C3 (n = 19)	0.39 (0.10–1.53)		0.41 (0.10–1.65)		0.69 (0.16–3.03)	
Per 10 μm	0.95 (0.91–1.00)	0.06	0.96 (0.91–1.02)	0.17	0.99 (0.94–1.05)	0.85
**Axial length**						
A1 (n = 119)	1.0 (ref)	P_trend_ = **0.01**	1.0 (ref)	P_trend_ = 0.06	1.0 (ref)	P_trend_ = 0.16
A2 (n = 41)	1.44 (0.66–3.14)		2.37 (0.94–5.99)		2.19 (0.85–5.65)	
A3 (n = 10)	2.07 (0.55–7.85)		2.40 (0.54–10.6)		1.82 (0.36–9.27)	
Per 1 mm	**1.42 (1.07–1.88)**	**0.01**	**1.61 (1.16–2.23)**	**0.005**	**1.58 (1.10–2.27)**	**0.01**

*CI* confidence interval, *OR* odds ratio, *PVD* posterior vitreous detachment, *ref* reference, *SD* standard deviation.

P for interaction by sex and age was 0.18 and 0.64, respectively, for the relationship between choroidal thickness and PVD; in contrast, it was 0.85 and 0.54 for the relationship betwen axial length and PVD, respectively. P values less than the statistically significant level (= 0.05) are marked in bold.

*Adjusted for age (in years) and sex.

^†^Adjusted for age (in years), sex, and axial length for choroidal thickness or choroidal thickness for axial length.

^‡^Choroidal thickness and axial length were classified into three groups based on the same range of each variable. The cut-off point was 51.0–186.9 μm (C1), 187.0–322.9 μm (C2), and 323.0–459.0 μm (C3) for choroidal thickness and 26.00–27.83 mm (A1), 27.84–29.67 mm (A2), and 29.68–31.50 mm (A3) for axial length.

The relationship of choroidal thickness and axial length with the PVD stages (stage 1 vs. stage 2 vs. stage 3 vs. stage 4) was analysed using logistic regression (Table 4). In the age-/sex-adjusted models, we observed statistically significant relationships of choroidal thickness and axial length with the increased PVD stages (P_trend_ < 0.01). Post-hoc power calculation using a sample size of 170 cases revealed 80% power to detect the following ORs for the second and third groups vs. the first group: 0.41 for choroidal thickness and 2.37 for axial length.

**Table 4:** Univariable and multivariable-adjusted logistic regression analyses of choroidal thickness in relation to the ordinal outcome of PVD stages (Stage 2 or 3 or 4 vs. 1) (n = 170)^‡^.

	Univariable OR (95% CI)	P value	Age-/sex-adjusted OR (95% CI)*	P value	Multivariable-adjusted OR (95% CI)^†^	P value
**Choroidal thickness**						
C1 (n = 47)	1.0 (ref)	P_trend_ = **0.005**	1.0 (ref)	P_trend_ = **0.009**	1.0 (ref)	P_trend_ = 0.41
C2 (n = 104)	**0.41 (0.21–0.79)**		**0.42 (0.21–0.87)**		0.76 (0.34–1.71)	
C3 (n = 19)	**0.28 (0.09–0.87)**		0.31 (0.09–1.01)		0.62 (0.17–2.20)	
Per 10 μm	**0.95 (0.91–0.98)**	**0.01**	0.96 (0.92–1.00)	0.06	0.99 (0.95–1.05)	0.95
**Axial length**						
A1 (n = 119)	1.0 (ref)	P_trend_ = **0.009**	1.0 (ref)	P_trend_ = **0.002**	1.0 (ref)	P_trend_ = **0.009**
A2 (n = 41)	1.74 (0.87–3.48)		**2.67 (1.23–5.80)**		**2.55 (1.15–5.63)**	
A3 (n = 10)	**4.32 (1.23–15.12)**		**5.16 (1.34–19.80)**		**4.29 (1.00–18.45)**	
Per 1 mm	**1.61 (1.23–2.10)**	**< 0.001**	**1.78 (1.32–2.40)**	**< 0.001**	**1.78 (1.29–2.45)**	**< 0.001**

*CI* confidence interval, *OR* odds ratio, *PVD* posterior vitreous detachment, *ref* reference, *SD* standard deviation.

P for interaction by sex and age was 0.05 and 0.87 for the relationship between choroidal thickness and PVD, respectively; in contrast, it was 0.58 and 0.37 for the relationship between axial length and PVD, respectively.

P values less than the statistically significant level (= 0.05) are marked in bold.

*Adjusted for age (in years) and sex.

^†^Adjusted for age (in years), sex, and axial length for choroidal thickness or choroidal thickness for axial length.

^‡^Choroidal thickness and axial length were classified into three groups based on the same range of each variable. The cut-off point was 51.0–186.9 μm (C1), 187.0–322.9 μm (C2), and 323.0–459.0 μm (C3) for choroidal thickness and 26.00–27.83 mm (A1), 27.84–29.67 mm (A2), and 29.68–31.50 mm (A3) for axial length.

To evaluate whether such a relationship differed according to age and sex, we added an interaction term to the age-/sex-adjusted models. We observed no significant interaction between axial length and PVD or the PVD stages (P > 0.30, Tables 3 and 4). However, a possible interaction was observed between choroidal thickness and sex, where the inverse association between choroidal thickness and advanced PVD stage was stronger in women than in men (P = 0.05, Table 4). Subsequently, we conducted sex-stratified analyses (Tables 5 and 6).

**Table 5:** Univariable and multivariable-adjusted logistic regression analyses of choroidal thickness in relation to the presence of complete PVD (vs. partial PVD) stratified by sex^‡^.

	Univariable OR (95% CI)	P value	Age-adjusted OR (95% CI)*	P value	Multivariable-adjusted OR (95% CI)^†^	P value
**Men (n = 94)**						
Choroidal thickness						
C1 (n = 41)	1.0 (ref)	P_trend_ = 0.51	1.0 (ref)	P_trend_ = 0.69	1.0 (ref)	P_trend_ = 0.97
C2 (n = 46)	0.59 (0.22–1.59)		0.60 (0.21–1.74)		0.78 (0.25–2.39)	
C3 (n = 7)	0.97 (0.16–5.69)		1.35 (0.19–9.53)		1.43 (0.19–11.08)	
Per 10 μm	0.99 (0.93–1.06)	0.79	1.00 (0.92–1.07)	0.91	1.02 (0.95–1.10)	0.57
Axial length						
A1 (n = 59)	1.0 (ref)	P_trend_ = 0.37	1.0 (ref)	P_trend_ = 0.17	1.0 (ref)	P_trend_ = 0.16
A2 (n = 30)	0.68 (0.13–3.43)		2.44 (0.82–7.27)		2.50 (0.82–7.60)	
A3 (n = 5)	1.25 (0.29–5.39)		1.71 (0.16–18.47)		1.89 (0.15–23.92)	
Per 1 mm	1.42 (0.96–2.10)	0.08	**1.55 (1.01–2.38)**	**0.04**	**1.63 (1.02–2.58)**	**0.04**
**Women (n = 76)**						
Choroidal thickness						
C1 (n = 30)	1.0 (ref)	P_trend_ = **0.04**	1.0 (ref)	P_trend_ = 0.10	1.0 (ref)	P_trend_ = 0.41
C2 (n = 37)	**0.27 (0.09–0.79)**		0.34 (0.10–1.24)		0.51 (0.13–2.05)	
C3 (n = 9)	0.33 (0.06–1.84)		0.31 (0.04–2.32)		0.54 (0.06–4.57)	
Per 10 μm	**0.91 (0.84–0.99)**	**0.03**	0.93 (0.85–1.01)	0.08	0.96 (0.87–1.06)	0.38
Axial length						
A1 (n = 60)	1.0 (ref)	P_trend_ = 0.26	1.0 (ref)	P_trend_ = 0.26	1.0 (ref)	P_trend_ = 0.78
A2 (n = 11)	0.95 (0.23–4.01)		0.95 (0.23–4.01)		1.49 (0.23–9.68)	
A3 (n = 5)	3.79 (0.58–24.75)		3.79 (0.58–24.7)		**1.18 (1.07–11.6)**	
Per 1 mm	1.50 (0.98–2.28)	0.06	1.65 (0.99–2.77)	0.06	1.45 (0.81–2.61)	0.22

*CI* confidence interval, *OR* odds ratio, *PVD* posterior vitreous detachment, *ref* reference, *SD* standard deviation.

In women, the cut-off point was 51.0–177.1 μm (C1), 177.2–298.7 μm (C2), and 298.8–459.0 μm (C3) for choroidal thickness and 26.00–27.86 mm (A1), 27.87–29.68 mm (A2), and 29.69–31.40 mm (A3) for axial length.

P values less than the statistically significant level (= 0.05) are marked in bold.

*Adjusted for age (in years).

^†^Adjusted for age (in years) and axial length for choroidal thickness or choroidal thickness for axial length.

^‡^Choroidal thickness and axial length were classified into three groups based on the same range of each variable.

In men, the cut-off point was 76.0–203.6 μm (C1), 203.7–330.3 μm (C2), and 330.4–459.0 μm (C3) for choroidal thickness and 26.00–27.82 mm (A1), 27.83–29.61 mm (A2), and 29.62–31.40 mm (A3) for axial length.

**Table 6:** Univariable and multivariable-adjusted logistic regression analyses of choroidal thickness in relation to the ordinal outcome of PVD stages (stage 2 or 3 or 4 vs. 1) stratified by sex^‡^.

	Univariable OR (95% CI)	P value	Age-adjusted OR (95% CI)*	P value	Multivariable-adjusted OR (95% CI)^†^	P value
**Men (n = 94)**						
Choroidal thickness						
C1 (n = 41)	1.0 (ref)	P_trend_ = 0.48	1.0 (ref)	P_trend_ = 0.66	1.0 (ref)	P_trend_ = 0.73
C2 (n = 46)	0.60 (0.25–1.41)		0.60 (0.24–1.48)		0.92 (0.35–2.45)	
C3 (n = 7)	0.99 (0.21–4.76)		1.44 (0.27–7.67)		1.88 (0.32–11.24)	
Per 10 μm	0.99 (0.94–1.05)	0.79	1.00 (0.94–1.06)	0.91	1.04 (0.98–1.11)	0.57
Axial length						
A1 (n = 59)	1.0 (ref)	**P**_**trend**_** = 0.04**	1.0 (ref)	**P**_**trend**_** = 0.02**	1.0 (ref)	**P**_**trend**_** = 0.01**
A2 (n = 30)	2.27 (0.93–5.51)		2.58 (0.99–6.69)		**2.82 (1.04–7.61)**	
A3 (n = 5)	3.69 (0.64–21.24)		5.59 (0.91–34.3)		**8.57 (1.14–64.68)**	
Per 1 mm	**1.69 (1.17–2.46)**	**0.006**	**1.80 (1.21–2.65)**	**0.003**	**1.99 (1.29–3.06)**	**0.002**
**Women (n = 76)**						
Choroidal thickness						
C1 (n = 30)	1.0 (ref)	**P**_**trend**_** = 0.005**	1.0 (ref)	**P**_**trend**_** = 0.01**	1.0 (ref)	P_trend_ = 0.19
C2 (n = 37)	**0.28 (0.11–0.72)**		0.39 (0.14–1.06)		0.60 (0.20–1.78)	
C3 (n = 9)	**0.17 (0.03–0.90)**		**0.18 (0.03–0.99)**		0.33 (0.05–2.01)	
Per 10 μm	**0.89 (0.83–0.96)**	**0.003**	**0.91 (0.85–0.98)**	**0.01**	0.94 (0.87–1.02)	0.13
Axial length						
A1 (n = 60)	1.0 (ref)	P_trend_ = 0.06	1.0 (ref)	P_trend_ = 0.06	1.0 (ref)	P_trend_ = 0.37
A2 (n = 11)	1.47 (0.43–4.98)		3.06 (0.79–11.84)		2.05 (0.50–8.50)	
A3 (n = 5)	5.98 (0.89–40.23)		4.23 (0.60–30.00)		1.90 (0.22–16.10)	
Per 1 mm	**1.63 (1.09–2.44)**	**0.02**	**1.76 (1.12–2.78)**	**0.02**	1.47 (0.89–2.44)	0.13

*CI* confidence interval, *OR* odds ratio, *PVD* posterior vitreous detachment, *ref* reference, *SD* standard deviation.

In men, the cut-off point was 76.0–203.6 μm (C1), 203.7–330.3 μm (C2), and 330.4–459.0 μm (C3) for choroidal thickness and 26.00–27.82 mm (A1), 27.83–29.61 mm (A2), and 29.62–31.40 mm (A3) for axial length.

In women, the cut-off point was 51.0–177.1 μm (C1), 177.2–298.7 μm (C2), and 298.8–459.0 μm (C3) for choroidal thickness and 26.00–27.86 mm (A1), 27.87–29.68 mm (A2), and 29.69–31.40 mm (A3) for axial length.

P values less than the statistically significant level (= 0.05) are marked in bold.

*Adjusted for age (in years).

^†^Adjusted for age (in years) and axial length for choroidal thickness or choroidal thickness for axial length.

^‡^Choroidal thickness and axial length were classified into three groups based on the same range of each variable.

Although the statistical power was limited, the inverse relationship between choroidal thickness and PVD stages was stronger in women than in men; the age-adjusted ORs of stage 4 PVD with the highest vs. lowest category were 0.18 (95% CI 0.03–0.99; P_trend_ = 0.01) for women and 1.44 (95% CI 0.27–7.62; P_trend_ = 0.66) for men (Table 6).

## Discussion

This study revealed that the prevalence of complete PVD was significantly higher in more advanced myopia, characterised by decreased choroidal thickness and increased axial length, in patients with high myopia (axial length ≥ 26 mm) aged 20–59 years old. After adjusting for age and sex, the PVD stage was significantly positively associated with axial length and inversely associated with choroidal thickness. Moreover, the association between choroidal thickness and PVD stage was suggested to be stronger in women than in men. These findings are clinically significant because they indicate that the morphological changes associated with myopia may be significant risk factors for the development of PVD in middle-aged adults.

To the best of our knowledge, this is the first study to explore the correlation between choroidal thickness and the advanced PVD grades in the early stages of myopia. The ordinal logistic regression analyses among highly myopic eyes showed a significant trend of decreased choroidal thickness with increased PVD stage, even after adjusting for age and sex. Consistent with those of previous studies^5,11,13^, our findings suggest that high myopia characterised by a thinner choroid is associated with earlier vitreous liquefaction and PVD at a younger age.

Although the pathophysiology of PVD has not yet been elucidated, the altered compositional and functional balance of the vitreous matrix (e.g. proteoglycans) and subsequent collagen aggregation have been suggested to contribute to vitreous liquefaction and weakening of post-basal vitreoretinal adhesion, thereby predisposing patients to PVD^21–24^. Moreover, proteoglycans are essential in maintaining the space between collagen fibrils, preventing them from self-aggregating^24^. Animal studies suggest existing synthesis and breakdown of vitreous collagen, even in adult eyes^25–27^. Notably, the important role of collagen metabolism has also been suggested in choroidal thickness changes in response to a myopic shift^28,29^. A study conducted on chick eyes reported that, compared to chicks wearing plano lenses, those wearing positive-powered lenses showed a significantly great amount of proteoglycan synthesis with choroidal thickening in response to myopic defocus, whereas those wearing negative-powered lenses showed a significantly small amount of proteoglycan synthesis with choroidal thinning in response to hyperopic defocus^29^. Additionally, studies have suggested that weakened vitreoretinal adhesion may occur because of the altered interactions of vitreous surface collagen fibrils and macromolecules on the inner surface of the retina^30–33^; however, the exact molecule involved in adhesion remains unknown. As the most highly vascularised tissue in the body, the choroid plays a significant role in maintaining ocular metabolism by supplying oxygen and nutrients to the retina^34^. Given that choroidal thickness is increasingly recognized as a proxy for choroidal blood flow^35^, it is plausible that decreased choroidal thickness may induce hypoxia in the retina and the adjacent structures^36^, thereby leading to altered collagen metabolism and subsequent vitreoretinal detachment. Although the role of collagen metabolism in conjugation with choroidal thickness and PVD in humans remains uncertain, we suggest a possibility that unbalanced collagen metabolism in a thinner choroid may exacerbate collagen aggregation, which commonly occurs in the early stage of PVD in high myopia.

The pivotal role of the choroid in myopic progression has been increasingly recognised^16–18^. By secreting numerous growth factors, the choroid may modulate the remodelling of the scleral extracellular matrix, thereby controlling ocular elongation^34^. Studies have also suggested that the choroid may intrinsically act as a mechanical barrier to the eye growth-related signalling molecules from the retina^34^. Indeed, animal studies of induced myopia have suggested that alterations in choroidal thickness preceded changes in axial length^28,37^. In a population-based study, choroidal thinning was associated with a myopic shift at an early stage, independent of axial length^19^. Given that a thinner choroid is a potential indicator for the myopic progression characteristics of disc and retinal lesions (e.g. a tilted disc or enlargement of peripapillary atrophy) in adolescents with high myopia^38^ and that changes in choroidal thickness are closely linked to the stretched retina, which can be a major cause of PVD^1–3^, we separately examined the relationship of choroidal thickness and axial length with PVD. However, in this study, a thinner choroid was not associated with PVD after adjusting for axial length and SE (Table 4). This may be attributed to the insufficient power to capture the relationship or to the fact that PVD may simply occur due to mechanical sequelae of globe elongation. Nevertheless, future longitudinal studies with large sample sizes are warranted to examine the complex interactions among choroidal thickness, axial length, and PVD.

In this study, the inverse relationship between choroidal thickness and PVD status was stronger in women than in men with highly myopic eyes at an early stage. A previous study suggested that the PVD stages were significantly higher in women than in men without high myopia^13^. In an age-matched, case–control study, a history of menopause (but not the duration of exposure to sex hormones) was reported as a significant risk factor for PVD, independent of age and myopia^39^. Considering that hormonal treatment has been shown to alter the concentration of vitreous hyaluronic acid in animal studies^40^, a stronger relationship between female sex and PVD observed in these studies may be partly attributed to vitreous liquefaction followed by perimenopausal hormonal changes. In fact, sudden hormonal changes in menopause are known to be risk factors for idiopathic macular holes^41^. Indeed, in our study, the majority (78%) of women with complete PVD (stage 4) were in their late 1940s or older, i.e. at the stage of initiation of perimenopausal changes. This may partly explain the stronger relationship of PVD to myopic shift in women. Nevertheless, further studies with larger sample sizes are warranted to confirm this finding.

The strength of our study included the use of advanced SS-OCT, which enabled us to visualise the posterior vitreous and precisely classify the PVD stages according to detailed criteria and with high validity.

However, this study also had several limitations. First, the sample size was relatively small, and no control group (stage 0 PVD) was included. Instead, we compared the prevalence of complete (stage 4) and partial PVD (stages 1–3) and showed a significant trend for the progression of PVD stages. Given that age and high myopia are significant risk factors for PVD^6^, collecting a sufficient and equal number of samples for each stage of PVD among patients with high myopia is difficult. Therefore, additional studies with larger samples, including various age groups, are warranted to confirm these findings. Second, we selected the eyes with better CDVA because eyes with low VA are likely to have cataracts, which significantly affects the quality of OCT images. There is a possibility that low VA could be caused by factors other than cataract (e.g., pathologic myopia); however, such cases were extremely limited because we excluded patients with ocular surgical histories or marked retinal disease. Because this study primarily aimed to explore the relationship between the myopic biometric parameters and the physiologic changes in normal PVD, we selected relatively healthy eyes (except those with high myopia). The majority of the examined eyes had high myopia rather than extremely high myopia or pathologic myopia (approximately 70% of the examined eyes had an axial length of 26.00–27.83 mm in this study). Therefore, further studies are necessary to examine this relationship in eyes with pathologic myopia. Third, considering that the choroidal thickness was measured from 8:00 a.m. to 5:00 p.m., we cannot eliminate the possibility that diurnal variation in the choroidal thickness might affect the results^42^. Nonetheless, we extracted information regarding the examination timing; additionally, we confirmed that the mean choroidal thickness did not differ based on the examination timing (morning vs. afternoon; the cut-off point was 12:30 AM, which was the midpoint of the examination time in this study). Although the sample size was limited, the inverse relationship between choroidal thickness and PVD stages was generally consistent, regardless of the examination timing: comparing the highest vs. lowest choroidal thickness groups, the ORs for the advanced PVD stage were 0.38 (95% CI 0.08–1.90; P_trend_ = 0.06) in subjects examined in the morning and 0.26 (95% CI 0.04–1.63; P_trend_ = 0.13) in those examined in the afternoon. Accordingly, we expect the effects of diurnal changes to be small in this study. Fourth, the retinal thickness was only measured at the fovea. Considering that the retinal thickness in highly myopic eyes varies at different regions^43^, further studies evaluating choroidal and retinal thickness at the peripapillary and subfoveal regions in relation to PVDs are warranted. Finally, owing to the cross-sectional nature of the study, we could not infer the causal relationship of axial length and choroidal thickness to PVD. In addition, this study did not reveal the mechanism underlying morphological changes related to myopia in PVD since the current static OCT images could not evaluate the functional change in the retinal status or altered interaction of the vitreoretinal interface. Therefore, future prospective studies exploring the retinal metabolic changes are warranted to reveal these relationships.

In conclusion, we observed a significant trend of decreased choroidal thickness, along with increased axial length, with an increased grade of PVD in patients with high myopia, particularly in women. While PVD is one of the most common physiologic changes of the human body, factors related to the actual process remain unknown due to the difficulty in identifying the initial stage^1–3^. Utilising advanced SS-OCT, our findings suggest that patients with high myopia having more pronounced myopic morphological changes (i.e. decreased choroidal thickness and increased axial length) should be highly prioritized and that fundus examinations with detailed OCT should be performed, even for young patients aged 20 years. Additionally, longitudinal observations on the relationship between morphological changes and PVD progression are warranted.

## Methods

### Setting and participants

The study protocol for this retrospective, cross-sectional study was approved by the Institutional Review Boards (IRBs) of the Hayashi Eye Hospital and Keio University School of Medicine (approval numbers, 2020-K-8 and 2020178) and followed the tenets of the Declaration of Helsinki. The Hayashi Eye Hospital and Keio University School of Medicine IRBs waived the need for written informed consent because of the retrospective nature of this study.

This study included consecutive patients who newly visited the clinic of the Hayashi Eye Hospital from 31 July 2019 to 30 September 2019. All the ophthalmic examinations were conducted on the same day when the study participants visited the clinic. One eye of each patient with better CDVA, or the right eye if both eyes had the same CDVA, was enrolled. The inclusion criteria were as follows: age between 20 and 59 years and axial length ≥ 26 mm. In contrast, we excluded patients who had a history of ocular surgery, inflammation or marked retinal disease, refused to participate in the study, had any difficulties undergoing the examinations, or those with missing SS-OCT information. However, we did not exclude eyes with abnormal PVDs characteristic of pathologic myopia, such as residual vitreous cortex after complete PVD, multiple PVDs, multi-layered PVDs (vitreoschisis), and thickened vitreous cortex adhering to retinal vessels at multiple points.

### Ophthalmic parameters

All ophthalmic examinations were performed by ophthalmic technicians who were unaware of the study purpose. The refractive spherical and cylindrical powers were measured without cycloplegia using an autorefractor instrument (KR-7100; Topcon, Tokyo, Japan). The SE value was calculated by adding the sum of the spherical power to half of the cylindrical power. The CDVA was measured on the same day the SS-OCT images were acquired. We converted the decimal value to the logarithm of the minimal angle of resolution for statistical analyses. The axial length of each eye was measured using SS-OCT (IOLMaster 700 Version 1.14; Carl Zeiss Meditec, Jena, Germany).

### SS-OCT imaging and assessment of choroidal thickness and PVD statuses

The average thickness of the subfoveal retinal and choroidal layers was measured using SS-OCT (PLEX Elite 9000 Version 1.7; Carl Zeiss Meditec). Details of the SS-OCT protocols have been previously described^5^. Briefly, this machine provided a fast-scanning speed of 100,000 A-scans/s, an axial resolution of 5.5 µm (in tissue), with a 1060-nm centre wavelength for visualising posterior ocular structures, including the vitreous and choroid.

Utilising the HD Spotlight 1 protocol with a 16-mm-wide section centred on the fovea, we acquired a horizontal image centred on the fovea and disc and a vertical image centred on the fovea. The scans were repeated 100 times in enhanced depth imaging mode and were averaged to generate the OCT images. Fast-Trac motion correction software was used for image acquisition. Retinal and choroidal thicknesses were measured at the fovea. The choroidal thickness was defined as the vertical distance from the retinal pigment epithelium line to the hyperreflective line behind the large vessel layers of the choroid (presumed to be the choroid-sclera interface). In contrast, retinal thickness was defined as the vertical distance from the internal limiting membrane to the interface between photoreceptor outer segments and retinal pigment epithelium. Experienced ophthalmic technicians enhanced the visualisation of the posterior vitreous by manually changing the image contrast and brightness and subsequently segmented each layer. If the line was blurred, the centre of the line was always traced and measured. OCT images with a signal strength index of less than 6 were excluded from the analyses.

One of the two experienced ophthalmic technicians acquired the OCT images and determined the PVD stages, as previously described^13^. When determining the PVD stages was difficult, both ophthalmic technicians confirmed the PVD stages. The inter-examiner and intra-examiner reproducibilities, which were given as kappa coefficients, were 0.9650 (95% CI 0.8975–1.000) and 1.000 (95% CI 1.000–1.0000), respectively (n = 40)^13^. According to the five-stage classification system^20^, we classified the PVD stages as stage 0 (no PVD), stage 1 (paramacular PVD), stage 2 (perifoveal PVD), stage 3 (vitreofoveal separation or peripapillary PVD), and stage 4 (complete PVD). In this study, no patients had stage 0 PVD. Subsequently, We categorised stage 1–3 PVDs as ‘partial PVD’ and stage 4 PVD as ‘complete PVD’.

### Statistical analysis

All significance tests were two-sided, and the significance level was set at α = 0.05. Analyses were performed using SAS (Version 9.4, SAS Institute, Cary, NC, USA). Baseline characteristics are shown as counts and proportions for categorical variables and means ± standard deviations for continuous values. Unpaired t-tests and χ^2^ tests were used to compare the differences between the partial and complete PVD groups. Given that age was a significant risk factor for PVD^4^, we stratified the patients by age into 20-year-old groups (e.g. 20 to < 40 years and 40 to < 60 years old) and conducted a partial correlation analysis to investigate any other ocular biometric factors related to PVD stages.

Our primary hypothesis test was to examine the relationship between choroidal thickness and axial length in the presence of PVD (partial vs. complete). We classified the choroidal thickness and axial length into three groups based on the same range of each variable to account for the external validity. Univariable-, age-/sex-, and multivariable- (including age, sex, and axial length for choroidal thickness/choroidal thickness for axial length) adjusted logistic regression analyses were conducted to obtain the ORs and 95% CIs. A trend test was conducted across the three categories of choroidal thickness and axial length by including the median value in each category. Additionally, we evaluated the relationship of choroidal thickness and axial length with the PVD stages (e.g. stage 1 [n = 101] vs. stage 2 [n = 13] vs. stage 3 [n = 10] vs. stage 4 [n = 46]) using univariable and multivariable-adjusted logistic regression analyses.

To evaluate whether the relationship of choroidal thickness and axial length with the presence of PVD (e.g. partial vs. complete) or the PVD stages may differ by age (i.e. the median age of 39 years) and sex, we tested the significance of various interaction terms in Wald tests added to each age-/sex-adjusted model.

## Data Availability

The datasets generated during and/or analysed during the current study are available from the corresponding author on reasonable request.
